# Ultra-high b-Value DWI in predicting progression risk of locally advanced rectal cancer: a comparative study with routine DWI

**DOI:** 10.1186/s40644-023-00582-7

**Published:** 2023-06-12

**Authors:** Guangwen Zhang, Ziliang Xu, Jianyong Zheng, Mian Wang, Jialiang Ren, Xiaocheng Wei, Yi huan, Jinsong Zhang

**Affiliations:** 1 Department of Radiology, Xijing Hospital, Fourth Military Medical University, No.127, Chang Le West Road, Xi’an, Shaanxi 710032 China; 2 Department of Gastrointestinal Surgery, Xijing Hospital, Fourth Military Medical University, Xi’an, Shaanxi 710032 China; 3 Department of Pharmaceuticals Diagnostics, GE Healthcare China, Beijing, 100176 China; 4 Department of MR Research, GE Healthcare China, Beijing, 100176 China

**Keywords:** Rectal neoplasms, Diffusion magnetic resonance imaging, Prognosis

## Abstract

**Background:**

The prognosis prediction of locally advanced rectal cancer (LARC) was important to individualized treatment, we aimed to investigate the performance of ultra-high b-value DWI (UHBV-DWI) in progression risk prediction of LARC and compare with routine DWI.

**Methods:**

This retrospective study collected patients with rectal cancer from 2016 to 2019. Routine DWI (b = 0, 1000 s/mm^2^) and UHBV-DWI (b = 0, 1700 ~ 3500 s/mm^2^) were processed with mono-exponential model to generate ADC and ADCuh, respectively. The performance of the ADCuh was compared with ADC in 3-year progression free survival (PFS) assessment using time-dependent ROC and Kaplan-Meier curve. Prognosis model was constructed with ADCuh, ADC and clinicopathologic factors using multivariate COX proportional hazard regression analysis. The prognosis model was assessed with time-dependent ROC, decision curve analysis (DCA) and calibration curve.

**Results:**

A total of 112 patients with LARC (TNM-stage II-III) were evaluated. ADCuh performed better than ADC for 3-year PFS assessment (AUC = 0.754 and 0.586, respectively). Multivariate COX analysis showed that ADCuh and ADC were independent factors for 3-year PFS (*P* < 0.05). Prognostic model 3 (TNM-stage + extramural venous invasion (EMVI) + ADCuh) was superior than model 2 (TNM-stage + EMVI + ADC) and model 1 (TNM-stage + EMVI) for 3-year PFS prediction (AUC = 0.805, 0.719 and 0.688, respectively). DCA showed that model 3 had higher net benefit than model 2 and model 1. Calibration curve demonstrated better agreement of model 1 than model 2 and model 1.

**Conclusions:**

ADCuh from UHBV-DWI performed better than ADC from routine DWI in predicting prognosis of LARC. The model based on combination of ADCuh, TNM-stage and EMVI could help to indicate progression risk before treatment.

**Supplementary Information:**

The online version contains supplementary material available at 10.1186/s40644-023-00582-7.

## Background

Rectal cancer is the main cause of cancer-related death and there is an apparent trend of increasing incidence for people younger than 50 years old [1]. Although the standardized treatment strategy was given, the prognosis varied significantly even for those who had the same tumor stage [2, 3]. Patients with a high risk of progression may require aggressive treatment, while low risk patients may benefit from conservative therapy [4]. Therefore, it is crucial to make a precise prediction about the progression risk with the aim of individualized treatment.

Diffusion weighted imaging (DWI) has been demonstrated to be a powerful modality in depicting tumor heterogeneity and perfusion by monitoring the movement of water molecules in vivo [5]. Previous studies have confirmed the correlation between the functional parameters of DWI and cancer characteristics, such as cellularity [6], angiogenesis, inflammation [7] and tumor stoma ratio (TSR) [8]. Furthermore, analysis of routine DWI involving prognosis prediction have been investigated recently in rectal cancer [9–11] and colorectal cancer [12, 13]. However, routine DWI has not performed satisfactorily and has exhibited controversial results in prognosis prediction of rectal cancer [14]. For example, a study [9] involving 128 patients with rectal cancer showed the ADC (b = 0, 1000 s/mm^2^) of tumor was not the independent factor for 3-year distant metastasis. While another study [11] including 61 patients with locally advanced rectal cancer found that the ADC (b = 0, 1000 s/mm^2^) of tumor was independently correlated with distant metastasis.

Recently, ultra-high b-value DWI (UHBV-DWI) is increasingly explored in relation to the cerebral system [15–17] and prostate cancer [18, 19] and has showed considerable potential in tumor grading and detection. In contrast, there is lack of research with respect to the investigation of UHBV-DWI in rectal cancer. Thus, in this study, UHBV-DWI was introduced to evaluate the progression risk of locally advanced rectal cancer (LARC) and compare with routine DWI.

## Methods

### Patients

The study was conducted in accordance with the Declaration of Helsinki. The institutional review board of our hospital approved this retrospective study and waived the requirement of informed consent for clinical data collection. Written informed consent was acquired for each MRI scan. Patients (n = 230) were consecutively recruited from November 2016 to May 2019 according to the following inclusive criteria: (1) pathologically confirmed rectal cancer; (2) multi-b value DWI performed at initial diagnosis. The exclusive criteria were: (1) non-adenocarcinoma and mucinous adenocarcinoma (n = 19); (2) received any cancer-related treatment before multi-b-value DWI (n = 5); (3) surgery was not performed during the process of disease (n = 8); (4) poor image quality of DWI (n = 13); (5) with other malignant tumors (n = 9); (6) TNM-stage I and IV (n = 45); (7) lost to follow-up (n = 19). Finally, 112 patients with TNM-stage II-III were enrolled for analysis. The process of patient selection is present in Figure S1. The outcomes in this study were 3-year progression free survival (PFS). The date of last follow-up was June 30, 2021.

### Multi-b value DWI acquisition

**Fig. 1:**
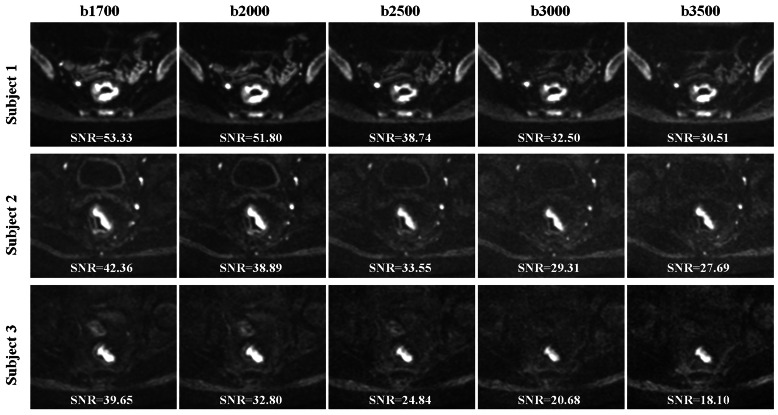
The tumor SNR at ultra-high b-value DWIs. The subject 1 (female, 26-year-old, TNM-stage III) represents highest SNR of first ten multi-b value DWI scans, subject 2 (male, 47-year-old, TNM-stage III) represents median SNR and subject 3 (male, 51-year-old, TNM-stage II) represents lowest SNR. Even with the lowest SNR, the tumor still could be clearly identified on b3500 DWI image

All patients received coronal and sagittal T2WI (TR/TE = 7355/136 ms), axial FRFSE T2WI (TR/TE = 5964/130 ms) with small FOV (220 × 220 mm), traditional DWI (b = 0, 1000 s/mm^2^) and multi-b-value DWI (b = 0 ~ 3500 s/mm^2^) on a 3.0 T MR scanner (Discovery MR750, GE Medical Systems), the detailed parameters of imaging sequence are shown in Table 1. At the beginning of this study, the imaging quality of first 10 multi-b value DWI scans were assessed and the ultra-high b-value DWI showed acceptable signal noise ratio (SNR) (Fig. 1). SNR was equal to the signal intensity of tumor divided by standard deviation of background noise. The average SNRs of tumor were 46.71 ± 5.38, 40.84 ± 5.43, 33.02 ± 4.50, 27.98 ± 3.76 and 25.07 ± 4.02 at b1700, b2000, b2500, b3000 and b3500 DWI images, respectively (Figure S2).

**Table 1:** Imaging sequence parameters

Parameters	Axial T2WI	Routine DWI	Multi-b value DWI
Scanning sequence	FRFSE	single-shot SE-EPI	single-shot SE-EPI
Repetition time (ms)	5964	4607	4607
Echo time (ms)	130	58.4	78.8
Field of view (mm)	220 × 220	400 × 320	400 × 320
Matrix	352 × 256	128 × 128	128 × 128
Intersection gap (mm)	0.3	0.5	0.5
Slice thickness (mm)	3.0	5.0	5.0
Number of slices	30	40	40
b-values	NA	0, 1000	0, 10, 20, 40, 80, 150, 200, 400, 800, 1000, 1200, 1500, 1700, 2000, 2500, 3000, 3500
Number of averages	2	1, 8	1 ~ 8*
Diffusion direction	NA	ALL	ALL
Number of directions	NA	3	3
Respiratory motion mitigation	Free-breathing	Free-breathing	Free-breathing
Flip Angle	90°	90°	90°
Acquisition time (min)	6:33	2:00	13:45

FRFSE, fast recovery fast spin echo; SE-EPI, spin echo-echo planar imaging; NA, not applicable. “*” indicates that the number of averages is 6 for b1700, b2000, b2500, b3000 and 8 for b3500 DWI acquisition

### ADC, ADCuh calculation and survival assessment

All multi-b value DWI were submitted to Firevoxel software (Copyright © 2021, New York University, USA) to perform registration with “AutoFocus motion correction” module before functional parameter calculation. The volumes of interest (VOI) were manually segmented on b1000 and b1700 DWI images by one abdomen radiologist (G.W.Z.) with 8-years’ experience using Firevoxel and then scrutinized by another senior radiologist (J.S.Z.) with 20-years’ experience. Routine DWI (b = 0, 1000s/mm^2^) was used to calculate ADC with VOI drawn at b1000 DWI and UHBV-DWI (b = 0, 1700 ~ 3500 s/mm^2^) was used to generate ADCuh with VOI drawn at b1700 DWI with mono-exponential model. The mono-exponential diffusion models above mentioned are as follows:

1. Mono-exponential diffusion model (Routine ADC)

$$ S\left(b\right)/{S}_{0}= {exp}\left(-b\cdot \text{ADC}\right), b=0, 1000 s/m{m}^{2}$$

2. Mono-exponential diffusion model (ADCuh) [15]:

$$ S(b)/{S_0}\, = \,exp( - b \cdot ADCuh),\,b\, = \,0\,\; and \; \ge 1700\,s/m{m^2}$$

${S}_{0}$ and $S\left(b\right)$ are the signal intensity obtained with the b-value equal to 0 s/mm^2^ and other given b-value diffusion weighted image. Traditional ADC represents the water diffusivity of diffusion-driven displacements. ADCuh derived from UHBV-DWI could characterize the transmembrane movement of water molecules via aquaporins[20]. Mean value of ADC and ADCuh were record for further analysis. The reproducibility of ADC and ADCuh between two different observers (G.W.Z. and Z.L.X.) was evaluated with intraclass correlation coefficient (ICC) based on the first 30 patients’ images.

The performance of ADC and ADCuh in predicting prognosis was explored with time-dependent receiver operator characteristic curve (ROC) and Kaplan-Meier curves. Univariate and multivariate COX proportional hazard regression model was used to perform survival analysis and construct prognostic models for 3-year PFS prediction with clinicopathologic factor and functional parameter of DWI. The time-dependent ROC, decision curve analysis (DCA) and calibration curve was used to evaluate the discrimination, net benefit and agreement of prognostic models respectively.

### Statistics

Statistical analyses were carried out with R software (version 4.1.2), and two-side *P* < 0.05 was considered statistically significant for all tests. R package “irr”, “survivalROC”, “survival”, “ggDCA” and “rms” was used to conduct ICC, time-dependent ROC, Kaplan-Meier curves, univariate and multivariate COX proportional hazard regression model, DCA and calibration curve respectively.

## Results

### Patient characteristics

A total of 112 patients with TNM-stage II-III were finally involved for analysis, including 72 males and 40 females. The mean age was 58.4 ± 12.5 (range: 26–88). The median follow-up time was 41 (range: 2–55) months. The 3-year PFS of the whole cohort was 76%. There were 11 (9.8%) patients who received surgery only, 101 (90.2%) patients took surgery and other therapy. Ninety-five (84.8%) patients showed negative mesorectal fascia (MRF) and 17 (15.2%) patients showed positive MRF. Eighty-three (74.1%) patients were negative EMVI and 29 (25.9%) patients were positive EMVI. The positive MRF was defined as the nearest distance between MRF and the tumor tissue was less than 1 mm, the tumor tissue included main tumor extension, extramural vascular invasion, tumor deposits or metastatic lymph nodes [21]. The positive EMVI was defined as apparent tumor signal within vessels with or without vessels expansion and irregular contour [22]. The status of MRF and EMVI were diagnosed based on the consensus of two abdomen radiologists on T2WI. The detailed characteristics of patients were summarized in Table 2.

**Table 2:** Patient baseline characteristics (n = 112)

Characteristics	Value
Age, years (Mean ± SD)	58.4 ± 12.5 (range, 26–88)
≥ 60, n (%)	53 (47.3)
< 60, n (%)	59 (52.7)
Gender, n (%)	
Male	72 (64.3)
Female	40 (35.7)
TNM-Stage#, n (%)	
II	37 (33.0)
III	75 (67.0)
T-stage, n (%)	
T2	14 (12.5)
T3	90 (80.4)
T4	8 (7.1)
N-stage, n (%)	
N0	37 (33.0)
N1	61 (54.5)
N2	14 (12.5)
MRF, n (%)	
negative	95 (84.8)
positive	17 (15.2)
EMVI, n (%)	
negative	83 (74.1)
positive	29 (25.9)
Treatment strategy, n (%)	
Surgery only	11 (9.8)
NAT + Surgery	8 (7.2)
Surgery + AT	66 (58.9)
NAT + Surgery + AT	27 (24.1)
Progression and Survival, n (%)	
Local recurrence	2 (1.8)
SODM	15 (13.4)
MODM	5 (4.5)
LRDM	3 (2.7)
Death	18 (16.1)
Differentiation, n (%)	
Well	11 (9.8)
Moderate	54 (48.2)
Poor	12 (10.7)
Unavailable	35 (31.3)

“#” indicates that TNM-stage of 35 (31.3%) patients were confirmed according to pelvic MRI and CT of chest and abdomen, as they had received neoadjuvant therapy before surgery. “Unavailable” denotes the data was not acquired. NAT, neoadjuvant therapy. AT, adjuvant therapy. SODM, single organ distant metastasis. MODM, multiple organ distant metastases. LRDM, Local recurrence and distant metastasis. MRF, mesorectal fascia. EMVI, extramural venous invasion

### Survival analysis with ADC and ADCuh

**Fig. 2:**
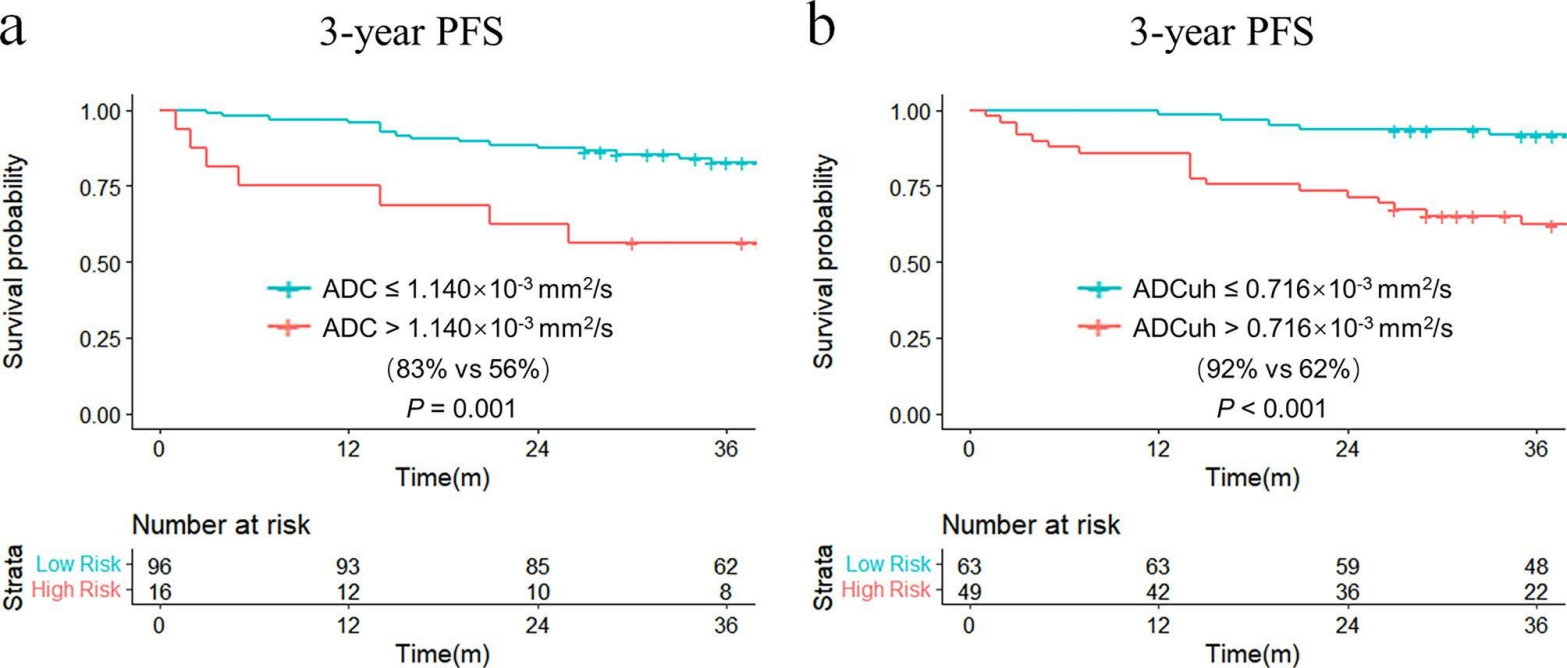
Kaplan-Meier curves of ADC and ADCuh. ADC and ADCuh could distinguish the 3-year PFS (**a**,**b**). The optimal cutoff values of ADC and ADCuh were acquired by using time dependent ROC (Fig. 3a)

**Fig. 3:**
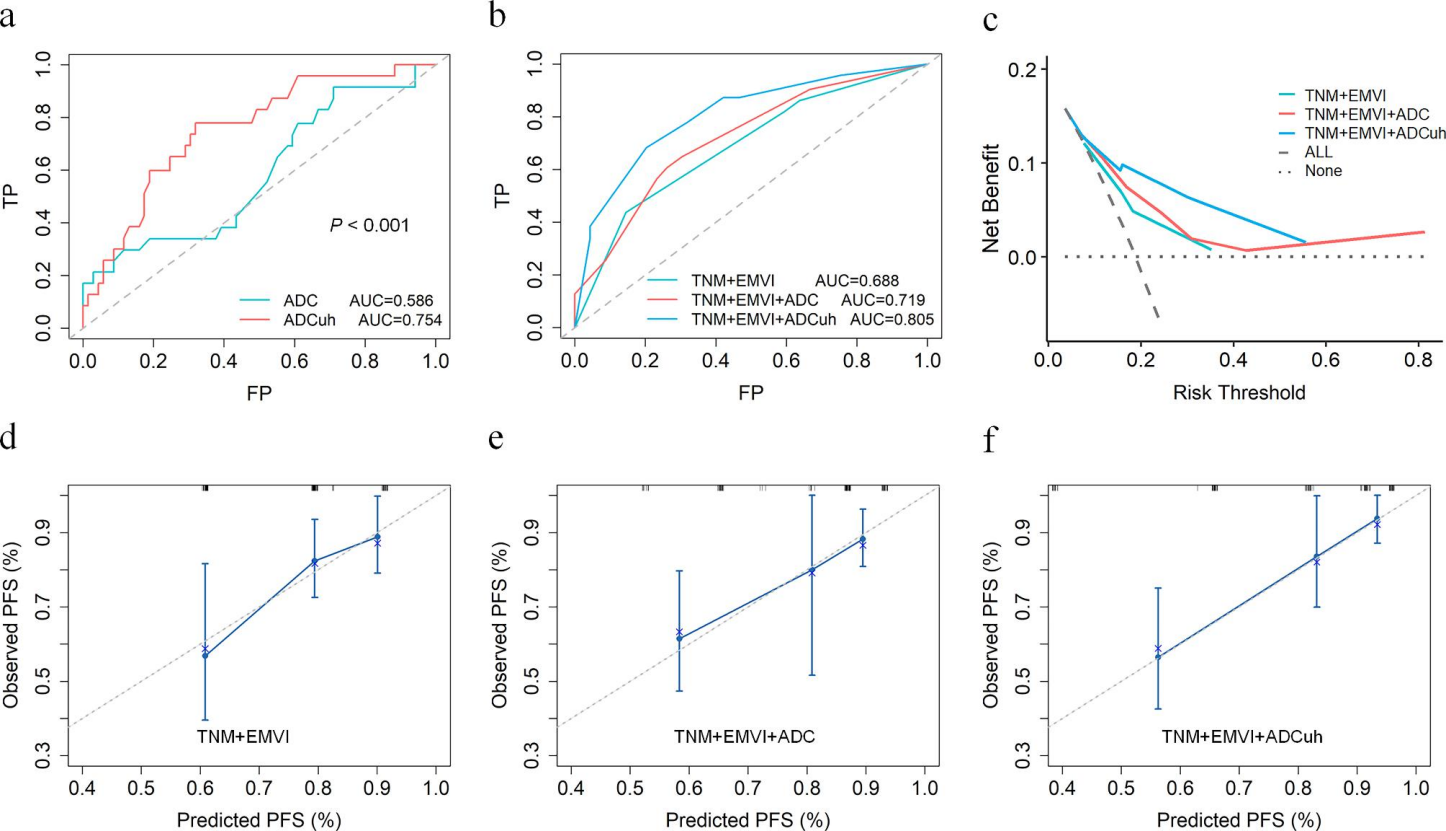
Survival analysis with time dependent ROC, decision curve analysis (DCA) and calibration curve. The ADCuh performed better than ADC in 3-year PFS evaluation (**a**). The prognostic model 3 (TNM + EMVI + ADCuh) was superior to model 2 (TNM + EMVI + ADC) and model 1 (TNM + EMVI) in 3-year PFS assessment (**b**). DCA showed that patients could have higher net benefit than model 2 and model 1 when risk threshold approximately ranged between 0.15 and 0.58 (**c**). Calibration curves (**d**-**f**) demonstrated better agreement of model 3 between predicted PFS and observed PFS than model 2 and model 1

**Fig. 4:**
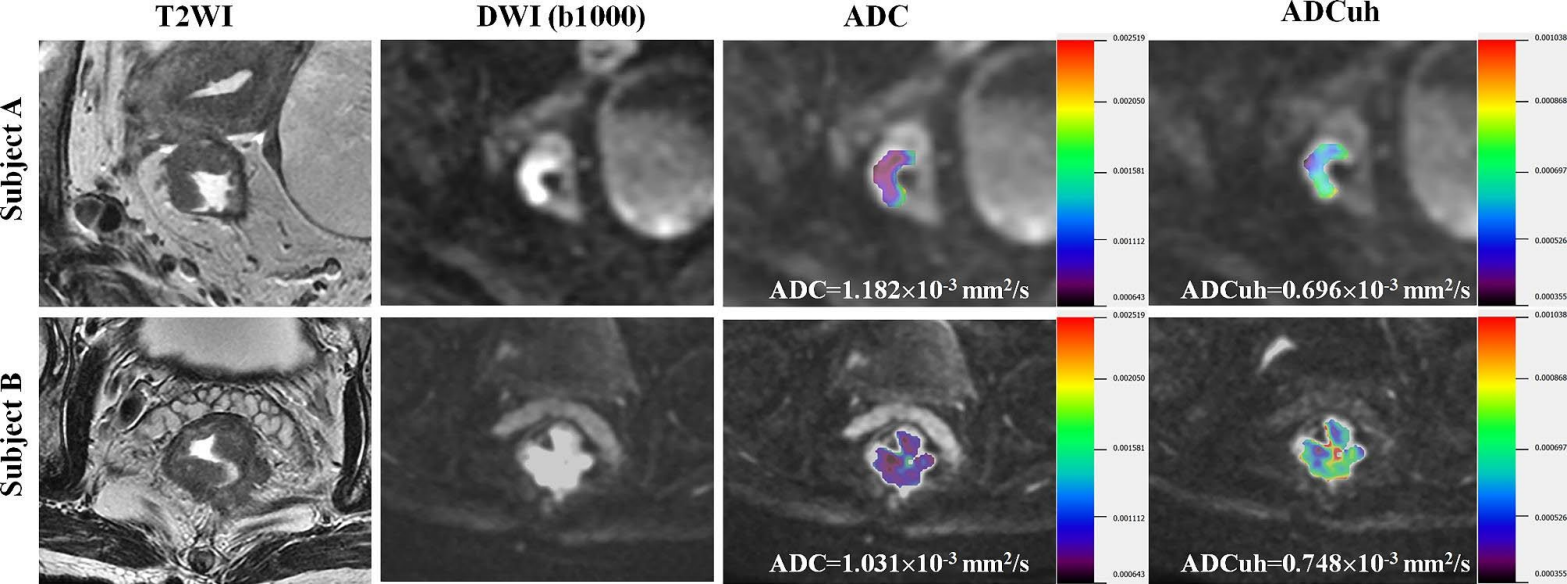
MR images of patients. Subject **A** (female, 76-year-old, TNM-stage II, EMVI- and MRF-) had no progression during follow-up. According to the cutoff values of ADC (> 1.140 × 10^− 3^ mm^2^/s) and ADCuh (> 0.716 × 10^− 3^ mm^2^/s), wrong prediction about progression for subject **A** will be made based on ADC value, while right prediction will be given based on ADCuh value. Subject **B** (male, 57-year-old, TNM-stage III, EMVI+, MRF+) had multiple organ distant metastases and died during follow-up. Wrong prediction for subject **B** will be made based on ADC value, in contrast, accurate prediction will be given based on ADCuh value

The ICC of ADC and ADCuh was 0.881 (95%CI: 0.522–0.971) and 0.933 (95%CI: 0.731–0.983), respectively, which demonstrated good reproducibility of ADC and ADCuh. By using the time dependent ROC analysis, the optimal cutoff values of ADC and ADCuh were 1.140 × 10^− 3^ mm^2^/s and 0.716 × 10^− 3^ mm^2^/s according to patients 3-year PFS. The Kaplan-Meier curves exhibited significant difference between the low ADC group and high ADC group in 3-year PFS (83% vs. 56%, *P* = 0.001, Fig. 2a) as same as ADCuh (92% vs. 62%, *P* < 0.001, Fig. 2b). Time-dependent ROC showed that ADCuh was superior to ADC in 3-year PFS assessment (AUC = 0.754 vs. 0.586, *P* < 0.001, Fig. 3a; Table 3). MR images of patients with proregression and without progression during follow-up was shown in Fig. 4.

**Table 3:** Time-dependent ROC for 3-year PFS assessment

Variate	Cutoff	AUC	Specificity	Sensitivity
ADC	1.140 × 10^− 3^ mm^2^/s	0.586	0.290	0.915
ADCuh	0.716 × 10^− 3^ mm^2^/s	0.754	0.681	0.778
TNM + EMVI*	0.930	0.688	0.855	0.437
TNM + EMVI + ADC *	1.105	0.719	0.739	0.607
TNM + EMVI + ADCuh*	1.571	0.805	0.797	0.683

“*” indicates the variate has no unit because it is a combined indicator generated with multivariate COX proportional hazard regression model. PFS, progression free survival. MRF, mesorectal fascia. EMVI, extramural venous invasion

### Prognostic model construction

When including age, gender, treatment strategy, TNM-stage, MRF, EMVI, ADC and ADCuh, univariate COX analysis (Table 4) revealed that TNM-stage, EMVI, ADC and ADCuh were related to 3-year PFS (all *P* < 0.05). Thus, multivariate COX analysis was performed with TNM-stage, EMVI, ADC and ADCuh for 3-year PFS and found that EMVI, ADC and ADCuh were the independent factors for 3-year PFS (Table 4).

In order to compare the prognostic value of ADCuh with ADC and clinicopathological factors, we constructed three prognostic models for 3-year PFS assessment, model 1 (TNM + EMVI), model 2 (TNM + EMVI + ADC) and model 3 (TNM + EMVI + ADCuh). The time dependent ROC indicated that model 3 has better performance than model 1 and model 2 (AUC = 0.805, 0.719, 0.688, respectively, Fig. 3b; Table 3). Decision curve (Fig. 3c) exhibited that patients might have higher net benefit than model 1 and model 2 when risk threshold approximately ranged between 0.15 and 0.58. Meanwhile, calibration curves (Fig. 3d-f) showed better agreement of model 3 between predicted PFS and observed PFS than other two models.

**Table 4:** Univariate and multivariate COX analysis for 3-year PFS.

Variate	Univariate			Multivariate	
	*P*	HR (95%CI)		*P*	HR (95%CI)
Age (> 60)	0.146	1.024 (0.992–1.058)			
Gender (female)	0.547	0.782 (0.351–1.741)			
Treatment (surgery plus other therapy)	0.645	0.753 (0.225–2.517)			
**TNM-stage** (III)	**0.046**	2.968 (1.018–8.656)		0.370	1.677 (0.542–5.191)
MRF (+)	0.115	2.099 (0.835–5.275)			
**EMVI** (+)	**0.021**	2.533 (1.148–5.589)		**0.015**	2.929 (1.236–6.939)
**ADC** (> 1.140 × 10^− 3^ mm^2^/s)	**0.002**	3.751 (1.616–8.707)		**0.025**	2.906 (1.145–7.378)
**ADCuh** (> 0.716 × 10^− 3^ mm^2^/s)	**< 0.001**	5.018 (2.002–12.580)		**0.005**	3.934 (1.505–10.283)

HR, hazard ratio; CI: confidence interval. PFS, progression free survival. MRF, mesorectal fascia. EMVI, extramural venous invasion. Age (≤ 60), gender (male), treatment (surgery only), TNM-stage (II), MRF (−), EMVI (−), ADC (≤ 1.140 × 10^− 3^ mm^2^/s) and ADCuh (≤ 0.716 × 10^− 3^ mm^2^/s) were as reference in Univariate and multivariate COX analysis

## Discussion

This study not only compared the performance of prognosis assessment of UHBV-DWI (b = 0, 1700 ~ 3500 s/mm^2^) with routine DWI (b = 0, 1000 s/mm^2^), but also demonstrated a combined model (TNM + EMVI + ADCuh) has good discrimination, net benefit and agreement for 3-year PFS prediction (AUC = 0.805). Herein, the ADCuh derived from UHBV-DWI performed better than ADC derived from routine DWI for 3-year PFS assessment in LARC (AUC = 0.754 vs. 0.586).

Though previous studies has demonstrated ADC was correlated with local recurrence or distance metastasis [9, 11] and disease-free survival [11], we found the performance of ADC was inferior to ADCuh in assessing PFS. The ADCuh is the functional parameter derived from UHBV-DWI according to the mono-exponential model in this study. Theoretically, when signal attenuation arrives into ultra-high b-value region, the sensitivity to smaller spatial scale enhances and enables DWI to explore tissue microstructure on complexity and heterogeneity more powerfully than routine DWI [23]. In fact, previous studies has showed that UHBV-DWI performed better than traditional DWI in tumor grading [16] and detection [18].

Interestingly, the patients with higher ADCuh or ADC was related to worse survival in this study, which was seemingly conflicting with traditional concept that lower ADC commonly indicated more aggressiveness characteristics of tumor and worse survival. On one hand, in our previous study, the ADCuh was found to be positively correlated with expression of AQP1 [24] which had been demonstrated to be an independently negative prognostic factor for stage II/III colon cancer [25], in other words, higher AQP1 expression indicated worse survival. On another hand, higher ADC might remind that apparent heterogeneity a quickly growing tumor harbored [26, 27], which was an important feature of more aggressive tumor [28]. Furthermore, higher ADC indicated tumors with more stromal infiltration and lower tumor stroma ratio (TSR) [8] which was definitely related to worse prognosis of rectal cancer [29].

Though the TNM-stage was not the independent factor for 3-year PFS in this study, which may be attributed to limited sample size and exclusion of patients with TNM-stage I and IV, it still was used to construct prognostic model with other independent factors (EMVI, ADC and ADCuh). According to the time-dependent ROC, DCA and calibration curve (Fig. 3b-f), the model 3 (TNM + EMVI + ADCuh) performed better than model 1 (TNM + EMVI) and model 2 (TNM + EMVI + ADC) in predicting 3-year PFS of LARC, which indicated that ADCuh could reveal additional information of tumor, except for stage, extramural venous invasion and cellularity.

The present study has some limitations that merit consideration. Up to now, there is no consensus about the b-value threshold for UHBV-DWI. Therefore, further research should focus on the optimal b-value range for investigating rectal cancer with UHBV-DWI. Higher b-value DWI needs longer acquisition time and is of low signal-noise ratio, poor spatial resolution and exacerbated image distortion. Though we performed good preparation before scanning and large number of averages (NEX), the impact of noise at high b-values DWI is not a trivial issue and needs to keep in mind when explaining the high b-value DWI data. Less b-values selection (two or three b values at high b-value DWI), maximum b value less than 3000 s/mm^2^ with 3.0T scanner and enough NEX (such as 8 for ultra-high b values DWI) may facilitate this technique being a regular practice. As non-Gaussian behavior is increasingly apparent with the increased b-values, non-Gaussian diffusion model, such as stretched exponential model (SEM) or diffusion kurtosis imaging (DKI), may be better to illustrate the signal attenuation of UHBV-DWI rather than the mono-exponential model we used here, which deserves to be explored in a future study. Though the combined model we constructed exhibited good performance, it still needs to be investigated and validated in additional datasets and future randomized controlled trials. Finally, auto-segmentation based on deep learning architecture, radiomics and other image modality, such as T2WI, are worthy of integrating into survival analysis in the next step.

## Conclusions

ADCuh based on UHBV-DWI is an independent prognosis factor for PFS of locally advanced rectal cancer and performed better than ADC from routine DWI. The model we constructed using a combination of ADCuh, TNM-stage and EMVI could be a promising tool for progression risk prediction before treatment.

## Electronic supplementary material

Below is the link to the electronic supplementary material.

Supplementary Material 1

Supplementary Material 2

## Data Availability

The datasets used and/or analyzed during the current study are available from the corresponding author on reasonable request.

## Abbreviations

ADC: Apparent diffusion coefficient derived from routine DWI
ADCuh: Apparent diffusion coefficient derived from ultra-high b-value DWI
DCA: Decision curve analysis
DWI: Diffusion weighted imaging
EMVI: Extramural venous invasion
LARC: Locally advanced rectal cancer
MRF: Mesorectal fascia
SEM: Stretched exponential model
SNR: Signal noise ratio
TSR: Tumor stroma ratio
UHBV-DWI: Ultra-high b-value DWI
