# Supplementary material for: Ultra-high b-Value DWI in predicting progression risk of locally advanced rectal cancer: a comparative study with routine DWI
Source: Cancer Imaging. 2023 Jun 12;23:59. doi: 10.1186/s40644-023-00582-7 (PMC10258936; doi:10.1186/s40644-023-00582-7)
Supplement: Supplementary file 1 — Supplementary Material 1 [file 40644_2023_582_MOESM1_ESM.docx]

**Supplementary materials**


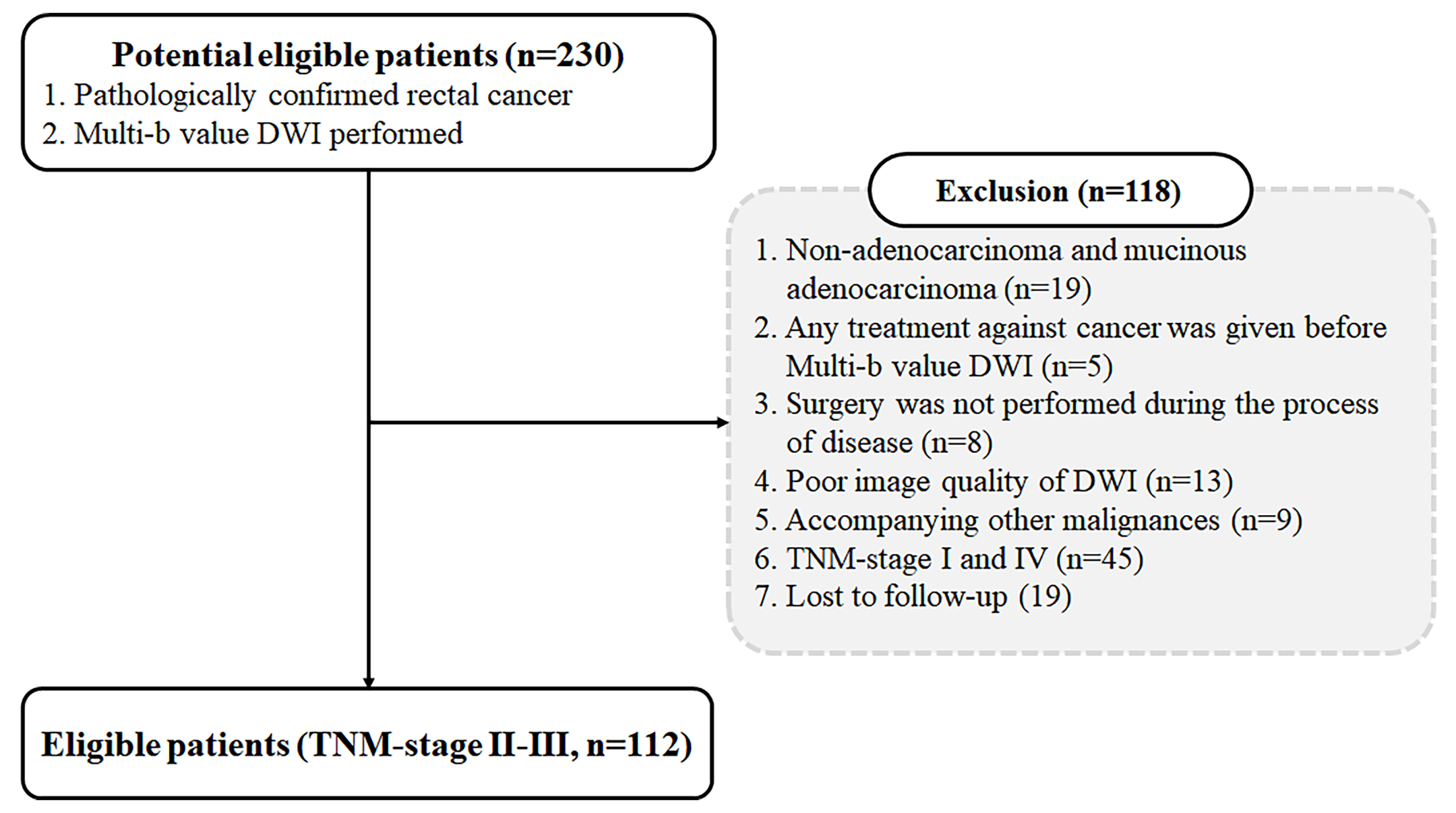


**Figure S1.** Flow chart of eligible participants selection.


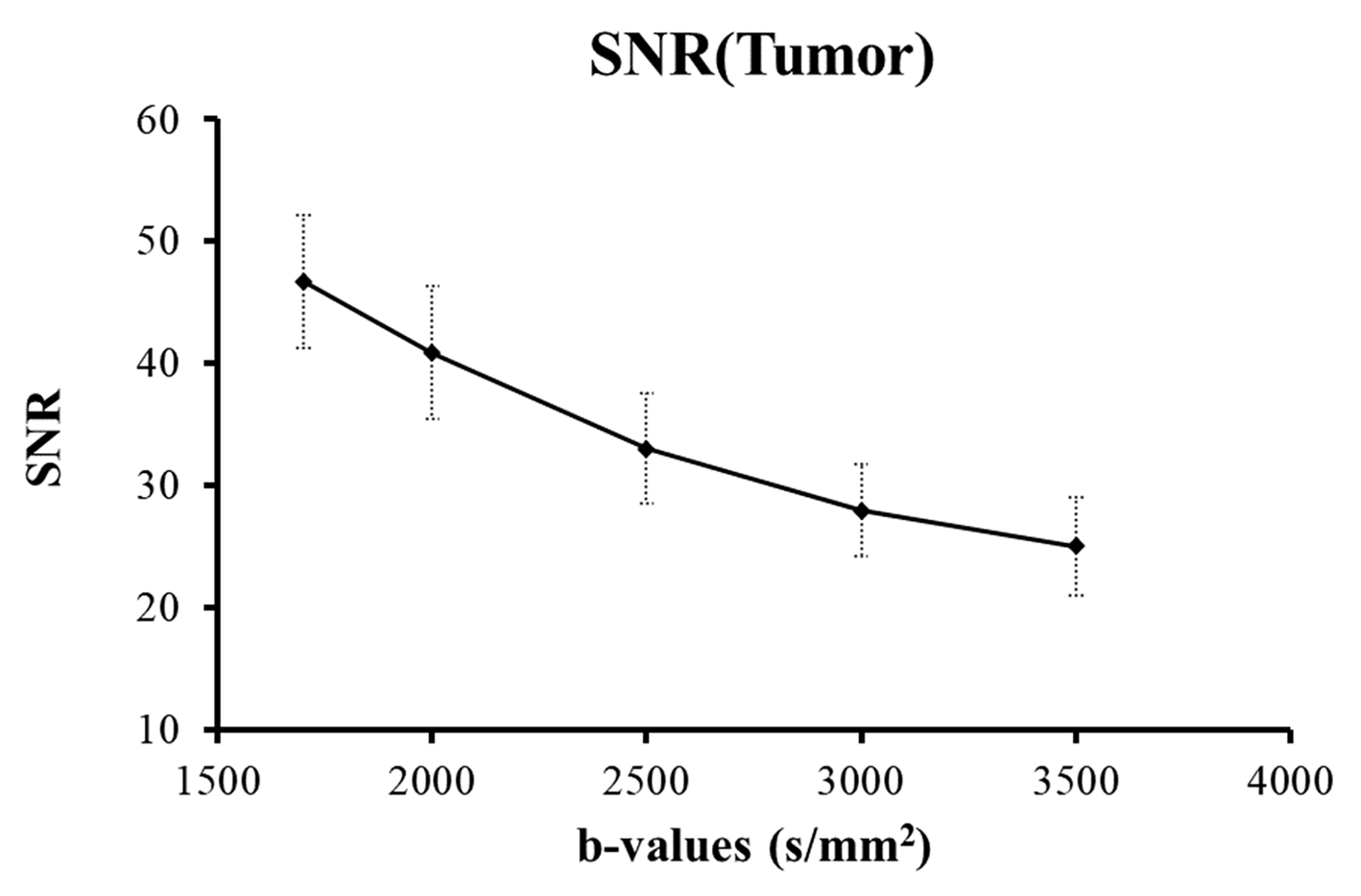


**Figure S2.** Tumor SNR at ultra-high b-value DWIs. The average SNRs of tumor were 46.71±5.38, 40.84±5.43, 33.02±4.50, 27.98±3.76 and 25.07±4.02 at b1700, b2000, b2500, b3000 and b3500 DWI images respectively.
