# Supplementary material for: Ultra-high b-Value DWI in predicting progression risk of locally advanced rectal cancer: a comparative study with routine DWI
Source: Cancer Imaging. 2023 Jun 12;23:59. doi: 10.1186/s40644-023-00582-7 (PMC10258936; doi:10.1186/s40644-023-00582-7)
Supplement: Supplementary file 2 — Supplementary Material 2 [file 40644_2023_582_MOESM2_ESM.docx]

Dear reviewer,

Thank you for your comments and suggestions. The point-by-point response was as follow, and all changes in revised manuscript were marked with red color.

**Comment 1：**Few concerns which I had - the use of high b values and processing the data in mono exponential formula may not be accurate. At higher b values the non-Gaussian pattern would set in. I am also concerned about the signal noise at high b values. Since the data is derived from multiple b values, the data could be used for IVIM or DKI processing. This would in turn give a comparative data that can be used quantitatively and qualitatively. This would make the study more relevant.

**Response：**Thanks for your professional suggestion. Yes, the signal attenuation of DWI at high b-values will deviate from Gaussian distribution. Non-Gaussian model, such as DKI, is more suitable for high b value DWI analysis. The diffusion of water at high b-values DWI were emphasized in this study rather than blood flow in capillary at low b-values DWI (e.g., b ≤ 200 s/mm^2^). In order to investigate the potential experience of high b-value DWI in tumor prognosis analysis, in this pilot study, mono-exponential analysis was conducted with b0 and high b values DWI (b = 1700~3500 s/mm^2^) images to derive ADCuh (termed ADCuh to differentiate from the conventional ADC). Different non-Gaussian diffusion models and b-values will be explored in our future study to optimize the b-value and diffusion model selection for possible clinical application.

The signal-to-noise ratio (SNR) at high b-value DWI were present in Figure 1 and Figure S2, in which the SNR was decreasing with the increasing b-values. However, the SNR was acceptable and tumor could be disentangled at high b-values, even though at b = 3500 s/mm^2^ DWI. Though we performed good preparation before scanning and large number of averages (NEX = 8 at b = 3500 s/mm^2^), the impact of noise at high b-values DWI is not a trivial issue and needs to keep in mind when explaining the high b-value DWI data.

The above limitations were discussed in last paragraph of discussion part (Page 17, line 14-45).

**Comment 2：**The statement that higher ADC values may indicate tissue heterogeneity needs to be bolstered with more references.

**Response：**Thanks for your advice. In this study, we found higher ADCuh or ADC values indicated worse survival and gave three possible reasons to explain this result. The second possible reason was that higher ADC might remind that apparent heterogeneity a quickly growing tumor harbored. We added relevant references in revised main text to support this possible reason (Page 16, line 34-39).

**Comment 3：**What was the authors' experience with metastatic nodes with regard to identification at high b values?

**Response：**With our initial experience, we found that radiomics features based on tumor ROI at b2000 DWI had the best performance in predicting positive nodes when compared with features at b1000 and b3000 DWI both in training set and testing set (Figure R1, below). It may be explained that high b-value DWI (b2000) has greater power in exploring tumor aggressiveness than routine DWI(b1000), while inferior performance of ultra-high b value DWI (b3000) may be attributed to low signal-noise ratio.

In our next study, we are going to compare the performance of ADC (b=0, 1000 s/mm^2^), ADC (b=0, 2000 s/mm^2^) and ADC (b=0, 3000 s/mm^2^) in detecting positive nodes based on node ROI, in which all nodes with diameter more than 5mm were included for analysis.

**Figure R1.** Lymph node prediction with radiomics features based on tumor ROI at different b-value DWIs. ADC value were calculated with b0 and b1000 DWIs. MutliB referred to a complex model based on combination of radiomics features of b1000, b2000, b3000 DWIs.

**Comment 4：**The noisy data may make Radiologists hesitant to use in regular practice. What would the authors suggest to make this technique a regular practice?

**Response：**The low SNR and long scanning time indeed hinder ultra-high b value DWI from regular practice. By reference from studies in prostate and our initial experience, good patient preparation, less b-values selection (two or three b values at high b-value DWI), maximum b value less than 3000 with 3.0T scanner and enough NEX (such as 8 for ultra-high b values) may facilitate this technique being a regular practice.
